# Network Analysis of Psychopathological Dimensions in Patients with and Without Suicidal Ideation

**DOI:** 10.3390/bs15070946

**Published:** 2025-07-14

**Authors:** Elena Huguet, Teresa Paniagua-Granados, Miriam Romero, Ignacio Fernández-Arias, Cristina Larroy

**Affiliations:** 1 Psychology Department, European University of Madrid, 28670 Madrid, Spain; teresa.paniagua@universidadeuropea.es (T.P.-G.);; 2 Personality, Assessment and Clinical Psychology, Complutense University of Madrid, 28223 Madrid, Spain; igfernan@ucm.es (I.F.-A.); clarroyg@ucm.es (C.L.)

**Keywords:** suicidal ideation, network analysis, psychopathological dimensions, anxiety, interpersonal variables, hostility

## Abstract

Knowing the configuration of psychopathological dimensions in patients according to suicidal ideation in an applied context is fundamental in clinical approaches. A cross-sectional and retrospective single-measurement study was carried out in 625 patients of the University Psychology Clinic of the Complutense University of Madrid (CUP-UCM) divided into two groups: with and without suicidal ideation. Network analysis was used as the main methodology. Anxiety and interpersonal variables appeared as core symptoms of the group with suicidal ideation, reinforcing theoretical models where the social component is a key part of present suicidal ideation. Even though the configuration of networks was not significantly different between the two groups, the severity of symptoms in the group with suicidal ideation was higher. In the predictive analyses, depressive symptoms, hostility, somatization, paranoid ideation, and psychoticism were independently associated with suicidal ideation. This result highlights the complex and multidimensional nature of suicide risk, which cannot be fully explained by depressive symptoms alone. Anxiety and interpersonal variables stand out as core symptoms with influence on others, which may guide clinicians in prioritization in therapeutic goals.

## 1. Introduction

Suicide, understood as death caused by self-directed harmful behaviour with the intention to die (Crosby et al., 2011), has become one of the major public health problems at national and international level. Already in 2014, the WHO raised the alarm on this issue and urged institutions to take specific measures and not to underestimate the risk (World Health Organization, 2014). According to the most updated figures from the National Institute of Statistics, in 2023 in Spain there were 4116 completed suicides, 5.6% more than in 2021, 1.6% more than in 2020, and 7.4% more than in 2019 (Instituto Nacional de Estadística, 2024).

Considering the entire suicide continuum, which also includes suicidal ideation and attempts of varying degrees of intensity and planning (Posner et al., 2011), the presence of suicidal ideation is a proven risk for suicide (De Leo et al., 2013; Hubers et al., 2018; Klonsky et al., 2016; Large et al., 2020). Across the board, the prevalence of suicidal ideation is higher than that of attempts (Castillejos et al., 2021) and 10 times higher than that of completed suicides (Nock et al., 2008).

In relation to the above-mentioned rising figures, and with the intention of focusing on suicide prevention, attempts have been made to identify the elements that are associated with suicidal behaviour. Although suicide is a complex phenomenon influenced by multiple factors—including demographic and sociosituational variables (Kim et al., 2016; Lorant et al., 2018; Nock et al., 2008; Sadock et al., 2015; Shah, 2011; Westefeld et al., 2015)—this study focuses specifically on the psychopathological dimensions (Nock et al., 2009; Nock et al., 2010) associated with suicidal ideation, as they are often the most proximal indicators of risk in clinical outpatient settings. Hopelessness (Franklin et al., 2017), despair (Glenn et al., 2022), psychological pain (Klonsky & May, 2015), impulsivity (Moore et al., 2022), and suicidality (Joiner, 2005; O’Connor & Kirtley, 2018) are some of the factors that clinicians should be aware of, as they are mechanisms related to the development and increased risk of suicidal behaviour.

Risk factors are known to transcend purely individual components, as they are also related to sociosituational factors such as periods of economic crisis, job instability, marital status or the absence of sociofamilial support networks (Blakely et al., 2003; Dehara et al., 2021; Milner et al., 2013a, 2013b). Therefore, the complexity of the phenomenon means that it should be approached from a global rather than an individualistic perspective (Al-Halabí & Fonseca-Pedrero, 2023).

Once it is known which factors are related to suicidal behaviour, it seems undeniable to ask the questions: is it sufficient to know these factors to address the current suicide crisis, and why are deaths still not preventable?

Klonsky et al. (2016) argued that the failure to predict completed suicide is due to a lack of knowledge about the processes underlying ideation and action, so theoretical explanatory models and related research should focus along these lines. Methodological approaches such as network analysis could overcome the tautological conceptualizations and reification of the common latent disorder model applied to psychology (Borsboom & Cramer, 2013). Psychopathological symptoms are conceived as networks of symptoms that evolve over time and are causally interrelated (Borsboom, 2017; Borsboom & Cramer, 2013; Fonseca-Pedrero, 2018; Fried et al., 2016), which is why this analytical approach has been applied to suicidal behavior in recent years (D. P. De Beurs et al., 2017; D. De Beurs et al., 2019; Fonseca-Pedrero et al., 2020b, 2022; Rath et al., 2019).

Most epidemiological studies have focused either on the general population or on specific psychiatric populations (psychotic patients, BPD or adolescents) (Ducasse et al., 2020; Fonseca-Pedrero et al., 2020a; Taylor et al., 2010; Yates et al., 2019). In fact, the Clinical Practice Guideline on the prevention and treatment of suicidal behaviour in Spain (2012) refers only to healthcare settings (primary care, mental health centers, and hospital emergency departments) for detection and intervention within the National Health System, without including the outpatient context outside the public health system. However, this context, a priori of a less urgent and/or serious nature than the hospital, may be the ideal setting for the detection of suicidal ideation at the beginning of its development, prior to its intensification or entry into the hospital circuit after attempts.

Thus, the main objective of this study is to explore the configuration and interrelationships of psychopathological symptoms in patients attending an outpatient clinical setting, according to the presence or absence of suicidal ideation. As a second objective, another aim is to analyze whether the relationships between dimensions are different in comparison with those for the group without suicidal ideation. For this purpose, we used a methodology based on psychopathological network analysis (Borsboom, 2017; Epskamp et al., 2018) and a predictive model that considers these dimensions.

It was hypothesized that symptom intensity would be higher in the group with suicidal ideation, with depressive and anxious symptomatology being the most central factors in the network. Furthermore, it was expected that there would be significant differences between the networks of both groups: depression would be more central in the network of people with ideation and there would be a positive relationship between depressive and anxious symptoms and suicidal ideation.

## 2. Materials and Methods

### 2.1. Participants

The sample was incidental composed of patients attending the University Psychology Clinic of the Complutense University of Madrid (CUP-UCM), which has been shown to have similar characteristics to other outpatient settings (Labrador et al., 2010). The participants in this study were all over 18 years of age, requested general psychological care, and signed the corresponding informed consent form.

The sample finally selected was 625 people, aged between 18 and 79 years, whose sociodemographic and clinical characteristics are summarized in Table 1.

Inclusion criteria required participants to be patients at the onset of the assessment process whose full clinical records were available in the archive of CUP-UCM. Participants had to be of legal age at the time of data collection and have completed the full assessment process. In addition, quantitative data from the required psychometric instruments had to be available, and all variables considered in the analysis had to be fully completed

Patients were excluded if the assessment responded to specific demands requiring alternative protocols, if therapy was conducted in a joint format that made it impossible to match individual patients to their corresponding questionnaires, or if the required psychometric data were incomplete or missing.

### 2.2. Instruments

- The Symptom Checklist-90-R (SCL-90-R) (Derogatis, 2001; González de Rivera et al., 2002). This self-administered instrument consists of 90 items that assess the presence of various psychopathological characteristics over the past seven days, specifically evaluating nine symptomatic dimensions and three global distress indices expressed in percentiles and T-scores. The items are structured on a Likert-type scale with five response options (0 to 4), where a higher score indicates greater symptom severity.
  Internal consistency for the instrument could not be assessed in our study, as item-level responses were not available—only the dimension scores were accessible. However, previous studies have reported adequate reliability for this version of the instrument. In similar clinical samples (Carrasco et al., 2003), the test has shown high internal consistency, both for the total scale (0.97) and for each of its subscales, which correspond to the nine pathological dimensions assessed: Somatization (0.87), Obsessive-Compulsive (0.87), Interpersonal Sensitivity (0.83), Depression (0.89), Anxiety (0.84), Hostility (0.84), Phobic Anxiety (0.88), Paranoid Ideation (0.80), and Psychoticism (0.76). In this study, T-scores of the nine psychopathological dimensions and the PSDI (Positive Symptom Total) were collected. For a detailed interpretation of each dimension, refer to the work of González de Rivera et al. (2002).
- To assess suicidal ideation, item 9 of the Beck Depression Inventory-II (BDI-II; Beck et al., 1996) was used, as has been done in previous research (Arria et al., 2009; Hom et al., 2018; Joiner et al., 2005; Pelizza et al., 2020). This particular item evaluates the presence of suicidal thoughts over the past two weeks using the following response format: 0 = I do not have any thoughts of killing myself; 1 = I have thoughts of killing myself, but I would not do it; 2 = I would like to kill myself; 3 = I would kill myself if I had the chance to do so. Following Taylor et al. (2015) and with the aim of dividing the sample into two groups, this item was dichotomized as follows: absence of suicidal ideation (level 0) and presence of suicidal ideation (levels 1, 2, and 3). In the present study, the full BDI-II scale showed excellent internal consistency (α = 0.91; ω = 0.91).

### 2.3. Procedure

The project was reviewed and approved by the Research Ethics Committee of CUP-UCM and complied with the requirements of the updated version of the Declaration of Helsinki (World Medical Association, 2013). All patients were informed and signed the informed consent, which outlined the general and specific objectives of the research and data collection process. The data were extracted from the clinical records filed at CUP-UCM. These records included, in addition to the patient’s clinical record, the initial data collection protocol and the psychometric tests administered.

Once it was verified that the subjects had given their consent to participate in the data collection, the elements relevant to this study were incorporated into the database. This database was subsequently stored in the general archive.

### 2.4. Data Analysis

A stability and accuracy analysis of the global network was conducted using the total sample (n = 625) as a reference. To avoid potential content overlap and enable a clearer conceptual and statistical distinction between depressive symptoms and suicidal ideation, we recalculated the Depression score using item-level data from the Beck Depression Inventory–II (BDI-II), excluding Item 9 (suicidal ideation). Specific undirected weighted psychometric networks were estimated for groups without suicidal ideation (n = 394) and with suicidal ideation (n = 203) using the bootnet (Epskamp et al., 2018) and qgraph (Epskamp et al., 2012) packages, following the standard guidelines suggested by Burger et al. (2022), Epskamp et al. (2018), and Epskamp and Fried (2018).

Using the bootnet package, a Gaussian graphical model (GGM) was estimated through the EBIC-glasso function, which applies the graphical least absolute shrinkage and selection operator (Graphical-LASSO or G-LASSO) for parameter regularization (Jones et al., 2018). For each network configuration, centrality measures, including strength centrality and expected influence, were computed. Strength centrality identifies the node with the highest number of connections (Borsboom & Cramer, 2013; Epskamp & Fried, 2018; Fonseca-Pedrero, 2018), while expected influence—defined as the sum of all edges connected to a node—determines which nodes exert the greatest influence within the network (Fonseca-Pedrero, 2022; Robinaugh et al., 2016). For each estimated network model, edge weights, standard errors, and *p* values were calculated to assess the strength and significance of the connections. These estimates are reported in supplementary tables in Appendix A (Table A3, Table A4 and Table A5), allowing for a detailed evaluation of the model parameters. Additionally, predictability and stability analyses were performed (Fonseca-Pedrero, 2017).

Finally, a comparative analysis between the two networks was conducted using the averageLayout function from the qgraph package (Epskamp et al., 2012), and the network comparison test (NCT) (Van Borkulo et al., 2022) was applied to assess differences between them. This permutation-based test evaluates network similarity across three dimensions: network structure (M), global network strength (S), and edge weight equality. The estimated edge weights, along with their standard errors and *p* values, are presented in supplementary tables in the Appendix A. These tables provide a detailed overview of the strength and statistical significance of the connections in the network models.

Finally, to identify psychopathological characteristics with predictive capacity for suicidal ideation, a binary logistic regression was performed. For the logistic regression analysis, the Depression variable was calculated using the total score from the BDI-II, excluding Item 9 (suicidal ideation), to avoid content overlap with the suicidal ideation predictor. Although the SCL-90-R also includes a Depression subscale, item-level data were not available for this measure, preventing the removal of its suicidal ideation item. Therefore, the BDI-II was used to ensure a cleaner separation between the constructs of depression and suicidal ideation in the analysis. Additionally, multicollinearity between the variables was assessed using the tolerance and variance inflation factor (VIF) indices, which help determine the extent of collinearity present, as high multicollinearity can lead to inflated variance in regression estimates.

## 3. Results

### 3.1. Descriptive and Comparative Statistics

The sample of patients with suicidal ideation (Table 2) consisted of 68.0% women (n = 138), with a mean age of 27.16 (SD = 10.366). The sample of the group without suicidal ideation was composed of 70.6% women (n = 278), with a mean age of 28.21 (SD = 11.91). There were no significant differences in sex (χ^2^(1) = 0.421, *p* = 0.516) or age (t(595) = 1.063, *p* = 0.288) between the two groups.

Statistically significant differences were found in each of the psychopathological dimensions of the SCL-90-R between the groups. The group with suicidal ideation exhibited more pronounced symptoms in each of the subscales of the SCL-90-R than the group without suicidal ideation.

### 3.2. Network Analysis of the Global Network of Patients (n = 625)

**Figure 1:**
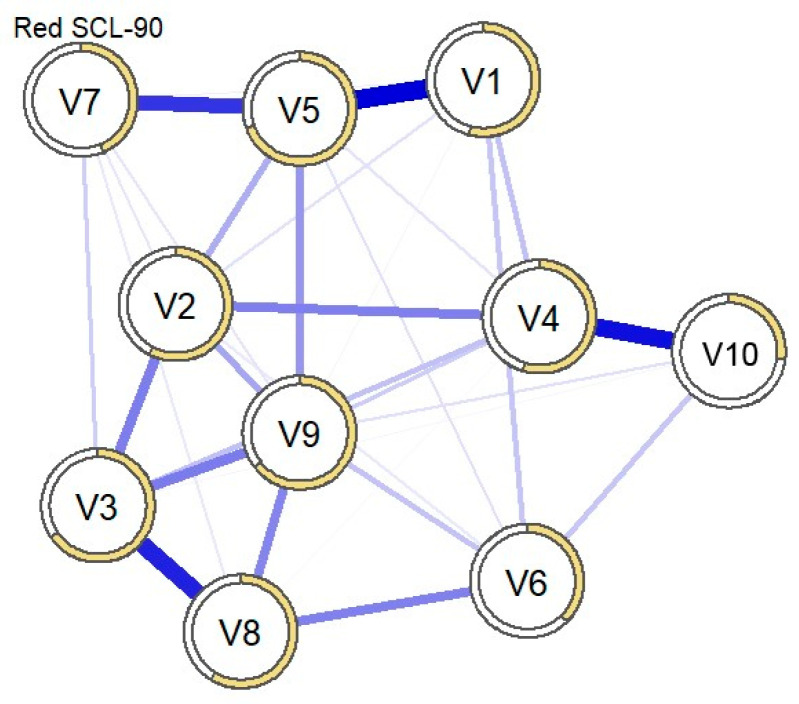
Network structure of the total sample using a Gaussian graphical model (n = 625). Note: V1 = Somatization, V2 = Obsessive-Compulsive, V3 = Interpersonal Sensitivity, V4 = Depression, V5 = Anxiety, V6 = Hostility, V7 = Phobic Anxiety, V8 = Paranoid Ideation, V9 = Psychoticism, V10 = Suicide.

Figure 1 shows the network of regularized partial correlations. Most of the edges represented positive correlations. The strongest relationships were found between “Somatization” and “Anxiety” (0.44), “Interpersonal Sensitivity” and “Paranoid Ideation” (0.38), and “Anxiety” and “Phobic Anxiety” (0.35). Additionally, relationships were observed between “Depression” and “Suicide” (0.32) and between “Hostility” and “Paranoid Ideation” (0.22).

**Figure 2:**
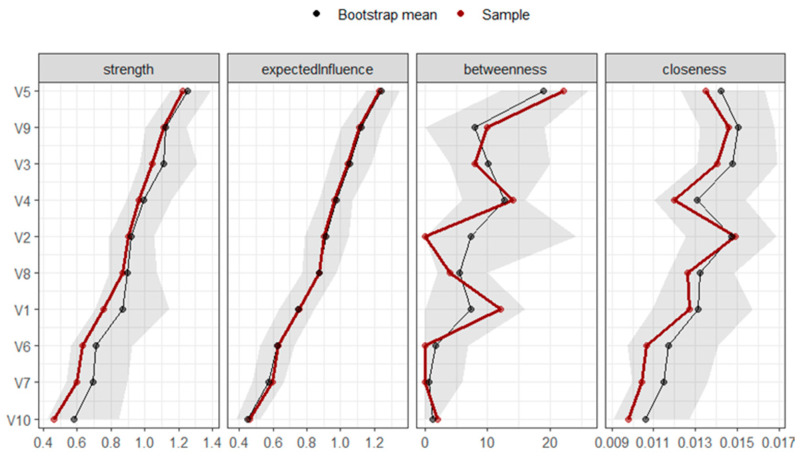
Graph of standardized centrality measures (n = 625). Note: V1 = Somatization, V2 = Obsessive-Compulsive, V3 = Interpersonal Sensitivity, V4 = Depression, V5 = Anxiety, V6 = Hostility, V7 = Phobic Anxiety, V8 = Paranoid Ideation, V9 = Psychoticism, V10 = Suicide.

Figure 2 shows graphs of the standardized centrality measures. The elements that showed the greatest expected influence were “Anxiety” (1.6), “Psychoticism” (1.03), and “Interpersonal Sensitivity” (0.26), with similar functioning in both the main sample and that derived from bootstrapping.

**Figure 3:**
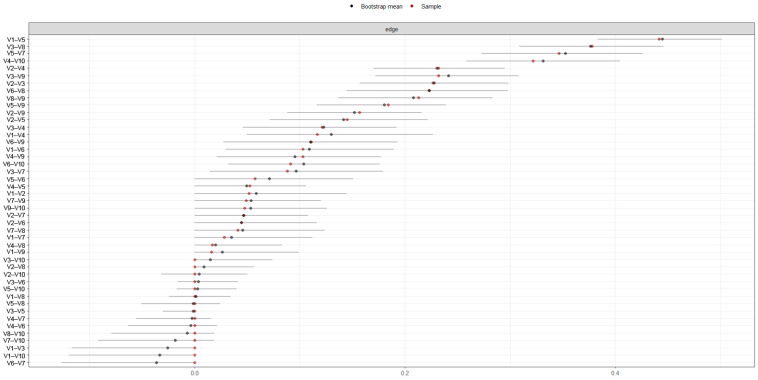
Bootstrap analysis of connections between nodes (n = 625). Note: V1 = Somatization, V2 = Obsessive-Compulsive, V3 = Interpersonal Sensitivity, V4 = Depression, V5 = Anxiety, V6 = Hostility, V7 = Phobic Anxiety, V8 = Paranoid Ideation, V9 = Psychoticism, V10 = Suicide.

Figure 3 presents the accuracy of the connections between all nodes using bootstrapping-based techniques (CI = 95%). The confidence intervals are wide, but we observed specific connections between nodes, and the estimated connections from the original sample were within the confidence interval, with an average predictability of 99.2%, ranging from 19.4% (V7 = Phobic Anxiety) to 66.9% (V4 = Depression).

**Figure 4:**
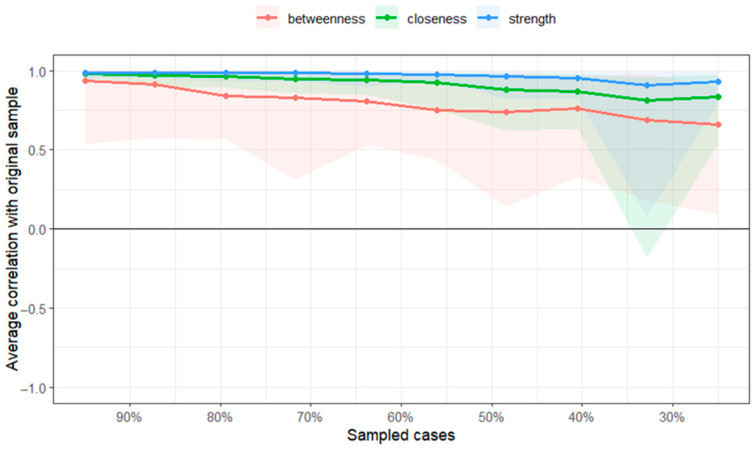
Stability of centrality indices (n = 625).

The network stability analysis (Figure 4) indicated that the strength centrality was moderate (CSstrength = 0.75), being above the cutoff point of 0.25 required for interpretation, considering the corresponding limitations (Burger et al., 2022), and above 0.50, ensuring that the centrality index has adequate stability (Epskamp, 2017; Hevey, 2018).

#### 3.2.1. Network Analysis of the Sample with Suicidal Ideation (n = 203)

**Figure 5:**
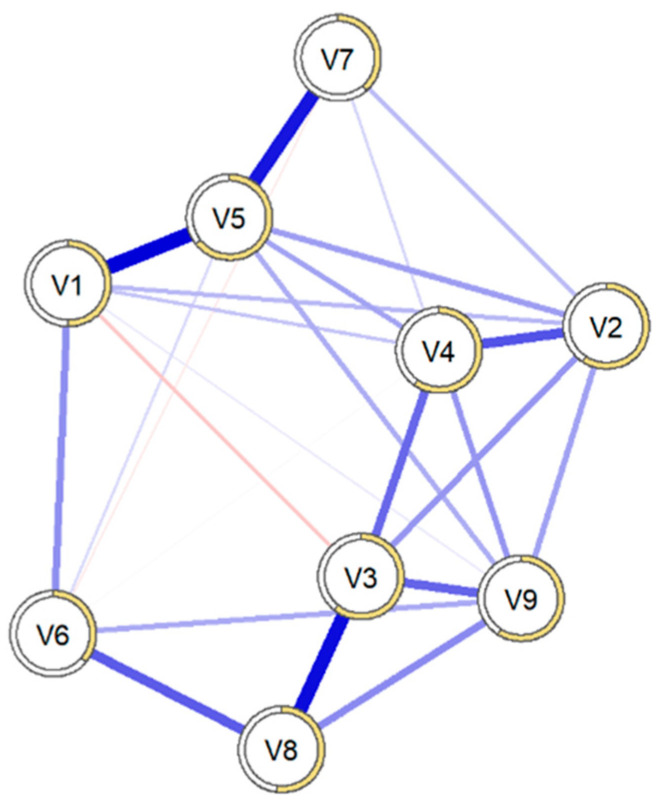
Structure of network configurations for patients with suicidal ideation (n = 203). Note: V1 = Somatization, V2 = Obsessive-Compulsive, V3 = Interpersonal Sensitivity, V4 = Depression, V5 = Anxiety, V6 = Hostility, V7 = Phobic Anxiety, V8 = Paranoid Ideation, V9 = Psychoticism.

The majority of edges represented positive correlations (Figure 5). The strongest relationships were found between “Interpersonal Sensitivity” and “Paranoid Ideation” (0.36), “Somatization” and “Anxiety” (0.39), “Anxiety” and “Phobic Anxiety” (0.35), “Hostility” and “Paranoid Ideation” (0.25), and “Depression” and “Obsessive-Compulsive” (0.26). There were also small negative correlations between “Interpersonal Sensitivity” and “Somatization” (−0.08) and between “Hostility” and “Phobic Anxiety” (−0.04).

**Figure 6:**
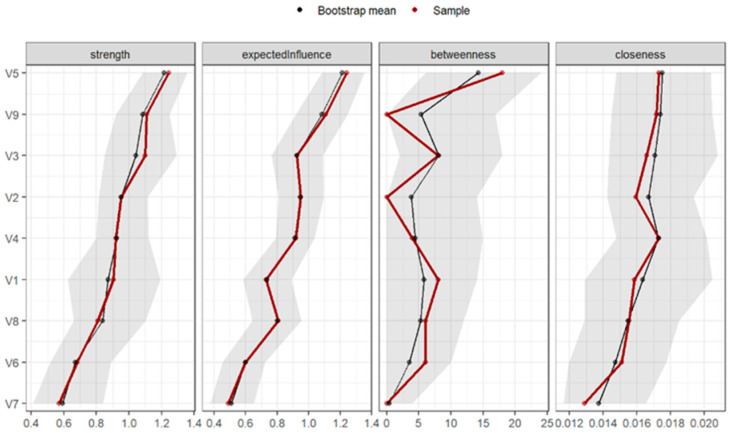
Graph of standardized centrality measures of networks of patients with suicidal ideation (n = 203). Note: V1 = Somatization, V2 = Obsessive-Compulsive, V3 = Interpersonal Sensitivity, V4 = Depression, V5 = Anxiety, V6 = Hostility, V7 = Phobic Anxiety, V8 = Paranoid Ideation, V9 = Psychoticism.

Figure 6 shows the plots of the standardised centrality measures. The dimensions with the greatest expected influence were “Anxiety” (1.50), “Psychoticism” (0.8), “Interpersonal Sensitivity” (0.83), and “Obsessive-Compulsive” (0.14). The elements with lower expected influence were “Phobic Anxiety” (−1.63), “Hostility” (−1.12), and “Paranoid Ideation” (−0.52).

The dimensions with the highest betweenness centrality (i.e., the greatest power of connection between nodes) were “Anxiety” (2.17), “Interpersonal Sensitivity” (0.42), and “Somatization” (0.42). Those with the lowest betweenness centrality were “Psychoticism” (−0.97), “Obsessive-Compulsive” (−0.97), and “Phobic Anxiety” (−0.97). In this case, the functioning was not as similar in the main sample and the bootstrap-derived sample.

Finally, the dimensions with the highest closeness centrality, indicating the greatest global power or access to the other nodes, were “Anxiety” (1.01), “Depression” (1.01), “Psychoticism” (0.91), and “Interpersonal Sensitivity” (0.06). The dimensions with the lowest closeness centrality were “Phobic Anxiety” (−2.16), “Hostility” (−0.57), and “Paranoid Ideation” (−0.25).

**Figure 7:**
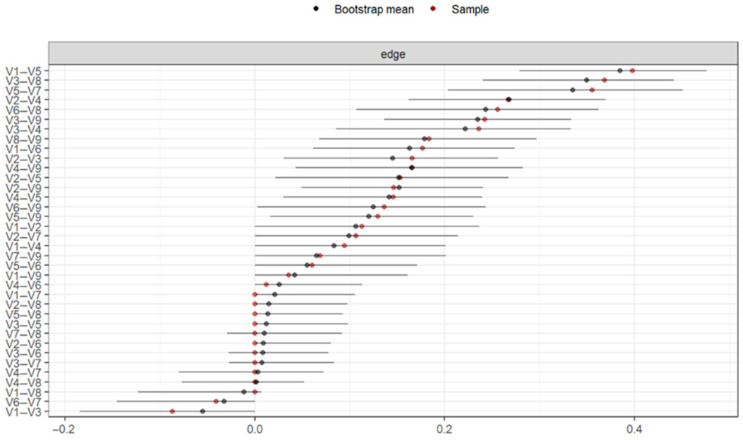
Bootstrap analysis of the connections between nodes (n = 203). Note: V1 = Somatization, V2 = Obsessive-Compulsive, V3 = Interpersonal Sensitivity, V4 = Depression, V5 = Anxiety, V6 = Hostility, V7 = Phobic Anxiety, V8 = Paranoid Ideation, V9 = Psychoticism.

The precision of the connections for all nodes was calculated using bootstrapping-based techniques (CI = 95%) as shown in Figure 7. The confidence intervals were wide, but there were specific connections between the nodes, and the estimated connections from the original sample fell within the confidence interval, with an average predictability of 53.5%, ranging from 36.4% (V6 = Hostility) to 64.5% (V5 = Anxiety).

The network stability analysis indicated that the centrality of strength is moderate (CSstrenght = 0.59), being above the threshold of 0.25, required for interpretation with the corresponding limitations, and above 0.50, ensuring that the centrality index has adequate stability.

#### 3.2.2. Network Analysis of the Sample Without Suicidal Ideation (n = 394)

**Figure 8:**
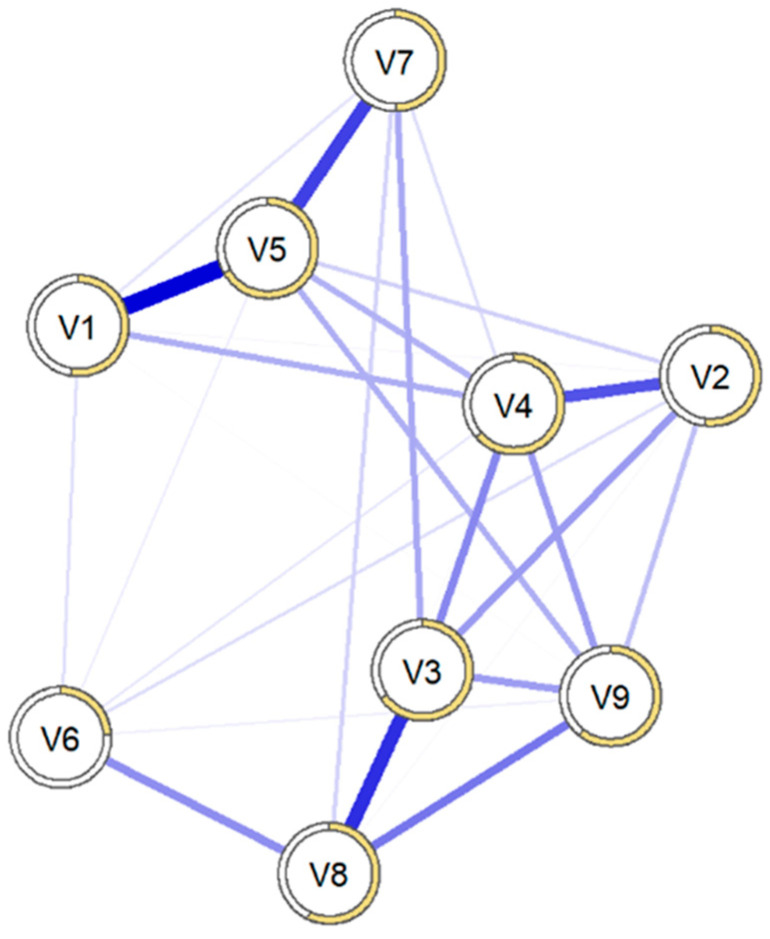
Structure of the network of the group without suicidal ideation using a Gaussian graphical model (n = 394). Note: V1 = Somatization, V2 = Obsessive-Compulsive, V3 = Interpersonal Sensitivity, V4 = Depression, V5 = Anxiety, V6 = Hostility, V7 = Phobic Anxiety, V8 = Paranoid Ideation, V9 = Psychoticism.

At a topographic level, as shown in Figure 8, the symptom grouping was similar to that of the group with suicidal ideation. In this case, all the edges represented positive correlations. The strongest relationships were found between “Interpersonal Susceptibility” and “Paranoid Ideation” (0.36), “Somatization” and “Anxiety” (0.44), “Anxiety” and “Phobic Anxiety” (0.33), “Hostility” and “Paranoid Ideation” (0.20), and “Depression” and “Obsessive-Compulsive” (0.29).

**Figure 9:**
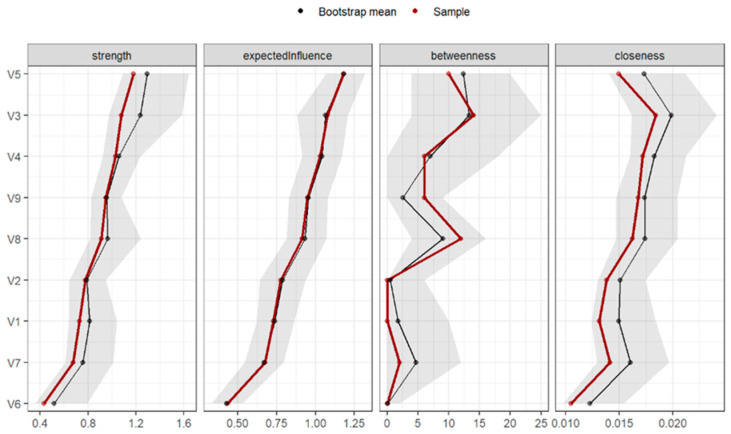
Centrality measure graphs of the networks of patients without suicidal ideation (n = 394). Note: V1 = Somatization, V2 = Obsessive-Compulsive, V3 = Interpersonal Sensitivity, V4 = Depression, V5 = Anxiety, V6 = Hostility, V7 = Phobic Anxiety, V8 = Paranoid Ideation, V9 = Psychoticism.

Figure 9 shows the standardized centrality measures, with similar performance in both the main sample and the bootstrapped sample. The elements that showed the greatest expected influence were “Anxiety” (1.37), “Interpersonal Susceptibility” (0.92), and “Depression” (0.72). The dimensions with the highest strength centrality were “Anxiety” (1.37), “Interpersonal Susceptibility” (0.92), and “Depression” (0.72).

The dimensions with the greatest strength centrality, as expected, were “Anxiety” (1.37), “Interpersonal Susceptibility” (0.92), “Depression” (0.72), “Psychoticism” (0.37), and “Paranoid Ideation” (0.21). The dimensions with the lowest centrality indices were “Hostility” (−1.85), “Phobic Anxiety” (−0.80), and “Somatization” (−0.58).

The dimensions with the highest betweenness centrality were “Interpersonal Susceptibility” (1.57), “Anxiety” (0.80), “Paranoid Ideation” (1.18), and “Depression” (0.08). The dimensions with the lowest betweenness centrality were “Somatization” (−1.01), “Obsessive-Compulsive” (−1.01), and “Hostility” (−1.01). In this case, and as happened in the group with suicidal ideation, the performance was not as similar in the main sample as in the bootstrapped sample.

Finally, the dimensions with the highest closeness centrality, those with the greatest global power or access to the rest of the nodes, were “Interpersonal Susceptibility” (1.42), “Depression” (0.79), “Paranoid Ideation” (0.52), and “Psychoticism” (0.74). The dimensions with the lowest closeness centrality were “Hostility” (−1.87), “Somatization” (−0.77), “Obsessive-Compulsive” (−0.49), and “Phobic Anxiety” (−0.34).

For a detailed comparison of centrality indices (expected influence, strength, closeness, and betweenness) across the three networks, see Appendix A, Table A2.

**Figure 10:**
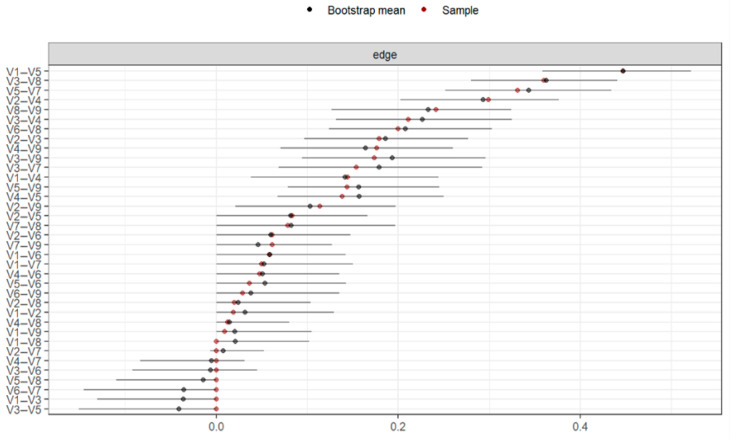
Bootstrap analysis of the connections between nodes (n = 394). Note: V1 = Somatization, V2 = Obsessive-Compulsive, V3 = Interpersonal Sensitivity, V4 = Depression, V5 = Anxiety, V6 = Hostility, V7 = Phobic Anxiety, V8 = Paranoid Ideation, V9 = Psychoticism.

In Figure 10, the precision of the connections between all the nodes is shown. There were specific connections between the nodes, and these were within the confidence interval. This indicates the precision with which we can assert that the connections between the nodes in our network were significantly different from zero, although with lower precision. The average predictability of the network was 54.7%, ranging from 36.4% (V6 = Hostility) to 64.5% (V5 = Anxiety).

The network stability analysis for patients without suicidal ideation reveals greater stability in the network (CSstrenght = 0.75), so the network can be interpreted with the same caution mentioned earlier, as it is above the reference value.

#### 3.2.3. Comparison of Networks Between Groups with and Without Suicidal Ideation

**Figure 11:**
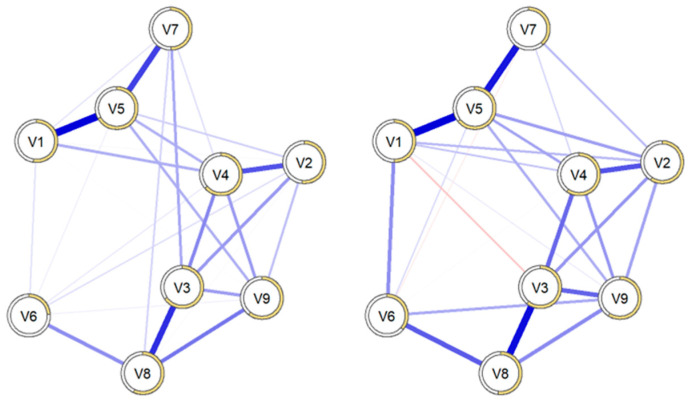
Network configuration structures of patients without suicidal ideation (left) and with suicidal ideation (right). Note: V1 = Somatization, V2 = Obsessive-Compulsive, V3 = Interpersonal Sensitivity, V4 = Depression, V5 = Anxiety, V6 = Hostility, V7 = Phobic Anxiety, V8 = Paranoid Ideation, V9 = Psychoticism.

Figure 11 displays both psychometric networks side by side to illustrate their overall configuration. In addition, bivariate comparisons of edge weights between the networks with and without suicidal ideation were also conducted and are presented in Appendix A, Table A1. In parallel, differences between the two network configurations regarding structure, strength, and equality were calculated (Table 3). The results first reflected that there was no difference in the structure of the networks, meaning both networks had the same number of connections between dimensions. There were also no differences in the strength of both networks, meaning the values between the nodes of both networks were similar.

Finally, the equality analysis, which evaluates specific connections between nodes, also indicated that there was no difference between the two networks.

### 3.3. Analysis of the Predictive Factors of Suicidal Ideation

Initially, no evidence of collinearity was found among the factors, given that all tolerance values exceeded 0.1 and the corresponding VIF scores remained below the threshold of 5 for all variables included (see Appendix A, Table A6).

The model fit indicated that at least one parameter of the model was significant (χ^2^(11) = 205,989, *p* < 0.01) and explained 39% (Nagelkerke R^2^ = 0.39) of the variance in suicidal ideation.

The results of the binary logistic regression are shown in Table 4. The dimensions of depression (β = 1.60; *p* = < 0.01) and hostility (β = 0.59; *p* = < 0.01) were the only variables that were independently associated with suicidal ideation.

## 4. Discussion

The main objective of this study was to analyze in an outpatient setting how psychopathological networks are configured in individuals with suicidal ideation and to explore the differences compared with those without suicidal ideation.

The initial bivariate contrasts confirmed that there were significant differences in symptoms between the two groups. As expected, patients with suicidal ideation presented more intense psychopathological symptoms at the start of therapy. This finding is consistent with much of the literature, which indicates a strong relationship between the presence of psychological disorders and the likelihood of experiencing suicidal ideation, attempts, and behaviors (May & Klonsky, 2016; San Too et al., 2019). Although the scores were consistently higher in all dimensions evaluated, depressive symptoms were clearly more pronounced in the suicidal ideation group (2.26 versus 1.40). However, what stood out the most were the significant differences in the dimensions of interpersonal susceptibility and hostility. Both dimensions have a strong relational component, suggesting that social factors play a key role in this phenomenon (Barker et al., 2022; Chu et al., 2017; Coppersmith et al., 2022; McClelland et al., 2020).

The results of the network analysis for the general sample showed that the most central symptoms for individuals in a clinical outpatient context were depression, anxiety, interpersonal susceptibility, and thoughts of social alienation. However, when analyzing only the network for individuals with suicidal ideation, the most central symptoms were anxiety, thoughts of social alienation, and interpersonal susceptibility. The most central node in terms of centrality strength and expected influence was anxiety, above other symptoms traditionally associated with suicidal behavior, such as depression (D. De Beurs et al., 2019; Franklin et al., 2017). This suggests that anxious symptoms are strongly connected to other symptoms and may represent key targets for clinical attention (McNally et al., 2015), although the cross-sectional nature of the data precludes assumptions about influence or directionality. This finding aligns with the logic of undirected network models, where changes in one symptom may be reflected in related symptoms; however, this study does not provide evidence of causal or directional effects. The fact that anxiety occupies such a central position further reinforces the idea that it is a highly disruptive process (Stanley et al., 2018) with wide-ranging implications across different areas of the individual (Olatunji et al., 2007), particularly in the population with suicidal ideation. In fact, anxious traits were also central in a recent network analysis study on suicidal ideation and emotional lability, aligning with the findings of this study. These authors (Núñez et al., 2020) proposed new hypotheses on how anxiety could lead to depression, rather than depression leading to anxious symptoms, as suggested by other studies (Cramer et al., 2010).

These findings connect directly with the current transdiagnostic view of psychological disorders. While the relationship with depressive disorders is clear (Beard et al., 2016; Casey et al., 2008), the psychological factors contributing to the development of suicidal ideation go beyond depressive symptoms (Klonsky et al., 2016). This was evident in the dimensions with high centrality, such as psychoticism and interpersonal susceptibility. Some items related to psychoticism, such as “Feeling lonely even when I am with others” and “Feeling always distant, with no sense of intimacy with anyone”, and items related to interpersonal susceptibility, such as “Feeling that others don’t understand me” and “Feeling inferior to others”, are considered clear risk factors for the development of suicidal ideation (Klonsky & May, 2015; Van Orden et al., 2010). These symptoms affect key risk factors such as sense of disconnection and loneliness, which again highlights the importance of the social component in this phenomenon.

This point is particularly relevant when interpreting psychometric tests in clinical settings, such as the SCL-90-R. These instruments are frequently used as screening tests in initial evaluations to help guide intervention, but suicidal risk can be overlooked if the connections between other dimensions not traditionally linked to suicide are not established.

The comparison between the psychopathological networks of individuals with and without suicidal ideation yielded unexpected conclusions. Statistically significant differences in the network configuration between the two groups were not observed. Contrarily to expectations, although patients with suicidal ideation had more intense symptoms across all psychopathological dimensions, the relationships between these dimensions were very similar in both groups.

This result suggests that, in clinical outpatient settings, although the initial severity of symptoms is greater in patients with suicidal ideation, the dimensions affected and the relationships between them are similar to those of individuals without ideation. This finding aligns with the perspective that suicide should not be viewed as a phenomenologically distinct phenomenon, but rather as a consequence of more intense distress, possibly experienced with greater anguish or a sense of being trapped (O’Connor & Kirtley, 2018). Moreover, this does not contradict the presence of general differentiating factors between the two populations, such as prior diathesis or vulnerability (Mann et al., 1999) or specific life events related to suicide and acquired capability (Joiner, 2005). These data reinforce the notion of dimensionality in the phenomenon, both in the suicidal continuum and its multiple manifestations (Barnow & Linden, 2000; Joiner et al., 2005; Sveticic & De Leo, 2012). Although the networks were similar in their structure and associative strength, there was evidence of systematically higher symptom intensity in patients with suicidal ideation.

To generate more evidence regarding the relationship between psychopathology and suicidal ideation, a binary logistic regression analysis was conducted, controlling for age and sex. Depressive symptoms, hostility, somatization, paranoid ideation, and psychoticism emerged as significant predictors of suicidal ideation, explaining 37% of the variance in the final model. The presence of depressive symptoms is consistent with previous studies (May & Klonsky, 2016; Tsai et al., 2021). However, the strongest risk factor was hostility, increasing the likelihood of suicidal ideation even after controlling for depressive symptoms. This suggests that hostility contributes uniquely to suicide risk, beyond the effects of depression. The fact that hostility showed such a strong effect highlights the role of interpersonal difficulties in suicidal ideation. This aligns with previous research linking hostility and aggressiveness to suicidal behavior (Gvion & Apter, 2011; Gvion & Levi-Belz, 2018; Lemogne et al., 2011; Menon et al., 2015), underscoring that suicide risk is not solely a matter of emotional distress but also involves perceived conflicts with others.

Beyond affective and interpersonal dimensions, the model also identified somatization, paranoid ideation, and psychoticism as relevant predictors, suggesting that additional forms of psychological dysregulation may elevate suicide risk through distinct mechanisms. Somatization may reflect unprocessed emotional pain manifested in physical symptoms, contributing to a sense of helplessness and unarticulated suffering that increases suicide vulnerability (Torres et al., 2021). Paranoid ideation, marked by mistrust and perceived threats from others, may lead to increased social withdrawal and heightened feelings of persecution, thereby reinforcing alienation and a sense of thwarted belongingness (Joiner, 2005). Finally, psychoticism—characterized by disorganized thinking and perceptual disturbances—may impair reality testing and intensify internal chaos, thereby diminishing coping capacity and increasing suicide risk (Yates et al., 2019).

While depressive symptoms remain clear predictors of suicidal ideation, these additional domains emphasize the multifaceted nature of suicide risk. Somatic complaints may mask underlying emotional distress, paranoid ideation can reinforce isolation, and psychotic features may erode one’s connection to reality—all factors that intersect with existing explanatory models of suicidal ideation and behavior (Joiner, 2005; Klonsky & May, 2015; O’Connor & Kirtley, 2018; Van Orden et al., 2010).

It is important to note that network and regression analyses address different, yet complementary, research questions. While network analysis focuses on the interrelations and connectivity between symptoms within a system—highlighting anxiety as a central symptom in this sample—regression models aim to identify which variables independently predict an outcome, in this case, suicidal ideation. The fact that hostility emerged as a significant predictor in the regression analysis does not contradict the centrality of anxiety in the network analysis; rather, it reflects that anxiety may be influential within the network structure, while hostility exerts a stronger predictive effect on suicidal ideation when controlling for other variables. Including both methods allows for a more comprehensive understanding of the psychopathological mechanisms underlying suicidal ideation.

Regarding the limitations of the study, the methodology had certain restrictions due to its developmental stage (Bringmann & Eronen, 2018; Brusco et al., 2019; Guloksuz et al., 2017; Jones et al., 2017; McNally et al., 2017), so the results should be interpreted with caution. Further analyses are needed to address issues related to the stability of the networks, as the current value was above the necessary threshold of 0.50 but below 0.70, which some authors consider adequate (Bringmann & Eronen, 2018).

The assessment of suicidal ideation at a single time point, without complementary information on prior or future ideation, limits the ability to explore this symptom’s temporal dynamics. This cross-sectional approach constrains interpretations related to the onset, persistence, or fluctuation of suicidal thoughts. Future longitudinal studies or pre-/post-treatment designs would allow for a more nuanced understanding of how suicidal ideation evolves in clinical outpatient populations.

A further concern is the mismatch between the timeframes of the instruments used, specifically, the BDI-II and the SCL-90-R, which assess symptoms over different reference periods. Although this discrepancy is relatively minor, it may have introduced additional measurement variability and complicate direct comparisons between the constructs evaluated by each instrument.

The gender composition of the sample also warrants consideration, with a predominance of women (66%). This imbalance may limit the generalizability of the results to populations with a higher representation of men. However, given the higher prevalence of suicidal ideation among women in epidemiological research, this distribution reflects patterns typically observed in clinical samples and is thus not unexpected in the present context.

A significant constraint lies in the absence of diagnostic data related to specific mental disorders, which prevented subgroup analyses by diagnostic category. This limits the interpretability of how symptom networks may differ according to underlying psychopathological profiles. Similarly, important clinical variables—such as history of suicide attempts, current psychopharmacological treatment, and substance use—were not collected. These factors are recognized as critical in assessing suicidal risk, and their absence reduces the depth and clinical applicability of the findings. Incorporating such variables in future research would enhance the contextualization and relevance of network structures.

An additional methodological issue involves the dichotomization of suicidal ideation based solely on item 9 of the BDI-II. While the use of this item is supported by prior research, and its predictive validity has been established (Green et al., 2015), this binary classification may lead to loss of nuance and restrict variability within subgroups. The two-week timeframe used may fail to capture individuals with relevant SI histories outside that window. Moreover, the case–control design applied may be subject to Berkson’s bias (de Ron et al., 2021), potentially affecting the representativeness and robustness of the estimated networks.

Future studies exploring differences in more specific subgroups could establish the dynamics between the dimensions addressed in this study. Furthermore, the implementation of network analysis based on the items of these dimensions could provide a deeper understanding of how they relate to each other and the directions of these relationships. In addition, incorporating complementary assessment tools may help differentiate psychopathological dimensions more precisely and further explore their unique contributions to suicide risk. This approach would ultimately refine the understanding of how specific psychological factors interact in the prediction of suicidal behavior.

Finally, this study provides updated evidence of the suicide phenomenon in clinical care contexts, serving as a starting point and a framework for future research on the subject, overcoming the previously mentioned limitations. This is the first study to use network analysis to examine the psychopathological symptoms of individuals with suicidal ideation in an outpatient clinical setting. Moreover, an important strength of this study is the use of the SCL-90-R, a widely employed instrument in routine clinical practice. This enhances the ecological validity of the findings, as the results are based on a tool frequently used in real-world consultations, making them more applicable to everyday clinical settings. Additionally, it is interesting to note that the distribution of means for the SCL-90-R dimensions in the present study closely resembled the study by Carrasco et al. (2003), highlighting the symptomatic similarities between clinical outpatient contexts, despite the time that has elapsed, and providing us with an updated reference of the main dimensions affected in a clinical outpatient context. Although the findings should be generalized cautiously to other populations, they have relevant clinical implications. Understanding how psychopathological symptoms relate to suicidal ideation, as well as the relevance of anxious and interpersonal symptoms in configuring the psychopathological networks of suicidal patients, can assist therapists in their evaluations/interventions and provide a therapeutic target for the “deactivation” of adjacent symptoms causing distress to the person. Furthermore, by using item 9 of the BDI-II, which assesses suicidal ideation within the past two weeks, the study deliberately focused on active or current suicidal ideation. This temporal frame enhances the clinical utility of the findings, as it allows for the identification of proximal risk factors and supports timely intervention in routine practice.

## Figures and Tables

**Table 1:** Sociodemographic and clinical characteristics of the sample (625).

Variables	n (%)/M (DT)
Age	27.76 (11.323)
Sex	
Female	435 (66.0%)
Male	190 (30.4%)
Nationality	
Spanish	565 (90.4%)
Other	60 (9.6%)
Occupational Status	
Student	386 (66.3%)
Employed	104 (17.9%)
Student and employed	60 (10.3%)
Unemployed	7 (1.2%)
Data not available	68 (4.3%)
Educational level	
Incomplete basic education	5 (0.9%)
Completed basic education	28 (4.8%)
Secondary education	309 (53.1%)
Professional education	34 (5.8%)
Bachelor’s degree	109 (18.7%)
Postgraduate degree	62 (10.7%)
Doctorate	7 (1.2%)
Data not available	71 (4.3%)
Marital Status	
Single	473 (81.3%)
Married/In a Stable Relationship	78 (13.4%)
Separated/Divorced	5 (0.9%)
Data not available	69 (4.3%)
Previous Psychological Treatment	
Yes	242 (41.6%)
No	308 (52.9%)
Data not available	75 (5.5%)
Depressive Symptoms (BDI-II)	21.59 (11.47)
Suicidal Ideation (Item 9 BDI-II)	394 (66.1%)
Absence of Suicidal Ideation	184 (30.8%)
Mild Suicidal Ideation	12 (2%)
Severe Suicidal Ideation	7 (1.2%)
Very Severe Suicidal Ideation	28 (4.5%)

Note. n = number of persons, % = percentage, M = mean, SD = standard deviation.

**Table 2:** Means, standard deviations, average ranks, and Mann–Whitney U tests of the sample with suicidal ideation (n = 203) and without suicidal ideation (n = 394).

Variables	Clinical Population with Suicidal Ideation (n = 203) M (SD)/Average Rank	Clinical Population Without Suicidal Ideation (n = 394) M (SD)/Average Rank	Mann–Whitney U	Z	Sig.(bil)
Somatization	1.29 (0.80)/355.74	0.90 (0.71)/269.77	28,473.5	−5.77	<0.01 ***
Obsessive-Compulsive	1.93 (0.78)/381.74	1.32 (0.79)/256.61	23,290.0	−8.37	<0.01 ***
Interpersonal Sensitivity	1.70 (0.79)/384.89	1.08 (0.83)/254.75	22,556.0	−8.74	<0.01 ***
Depression	2.26 (0.70)/416.58	1.40 (0.73)/238.89	16,123.0	−11.96	<0.01 ***
Anxiety	1.53 (0.82)/371.56	1.02 (0.72)/261.61	25,261.0	−7.38	<0.01 ***
Hostility	1.32 (0.96)/382.77	0.68 (0.67)/255.84	22,985.5	−8.55	<0.01 ***
Phobic Anxiety	0.79 (0.75)/356.82	0.47 (0.61)/269.21	28,254.5	−5.93	<0.01 ***
Paranoid Ideation	1.24 (0.81)/367.21	0.78 (0.71)/263.85	26,143.5	−6.95	<0.01 ***
Psychoticism	1.14 (0.65)/392.29	0.62 (0.55)/250.94	21,053.5	−9.50	<0.01 ***

Note. M = mean, SD = standard deviation; *** *p* < 0.001, Z = Z score, Sig.(bil) = Asymptotic two-tailed significance.

**Table 3:** Results of the network comparison test between groups.

Contrasts	Statistic	Significance
Network Structure	M = 0.15	0.43
Network Strength	S = 0.26	0.65

**Table 4:** Binary logistic regression (n = 625).

Model 1	β	Error	df	Sig.
Sex	−0.159	0.240	1	0.507
Age	0.017	0.010	1	0.091
Somatization	−0.438	0.204	1	0.032 *
Obsessive-Compulsive	−0.043	0.188	1	0.819
Interpersonal Susceptibility	0.133	0.204	1	0.516
Depression	0.110	0.014	1	<0.01 ***
Anxiety	0.056	0.228	1	0.804
Hostility	0.698	0.154	1	<0.01 ***
Phobic Anxiety	0.004	0.198	1	0.982
Paranoid Ideation	−0.422	0.204	1	0.039 *
Psychoticism	−3.864	0.272	1	0.027 *

Note: * *p* < 0.05 *** *p* < 0.001, β = beta, Sig. = significance level.

## Data Availability

The data presented in this study are available on request from the corresponding author. The data are not publicly available due to privacy and ethical restrictions.

## Appendix A

**Table A1:** Bivariate correlations of the networks of patients without suicidal ideation (above the diagonal, outside brackets) and with suicidal ideation (above the diagonal, in brackets).

Variables	1	2	3	4	5	6	7	8	9
1. Somatization	1	0.46 *** (0.55 ***)	0.39 *** (0.33 ***)	0.56 *** (0.52 ***)	0.72 *** (0.66 ***)	0.35 *** (0.44 ***)	0.51 *** (0.40 ***)	0.41 *** (0.27 ***)	0.49 *** (0.47 ***)
2. Obsessive-compulsive	0.46 *** (0.55 ***)	1	0.63 *** (0.62 ***)	0.68 *** (0.68 ***)	0.54 *** (0.65 ***)	0.39 *** (0.39 ***)	0.45 *** (0.48 ***)	0.53 *** (0.45 ***)	0.58 *** (0.63 ***)
3. Interpersonal sensitivity	0.39 *** (0.33***)	0.63 *** (0.62 ***)	1	0.66 *** (0.64 ***)	0.47 *** (0.49 ***)	0.36 *** (0.42 ***)	0.54 *** (0.35 ***)	0.70 *** (0.66 ***)	0.67 *** (0.67 ***)
4. Depression	0.56 *** (0.52 ***)	0.68 *** (0.68 ***)	0.66 *** (0.64 ***)	1	0.63 *** (0.60 ***)	0.39 *** (0.43 ***)	0.48 *** (0.40 ***)	0.55 *** (0.45 ***)	0.65 *** (0.63 ***)
5. Anxiety	0.72 *** (0.66 ***)	0.54 *** (0.65 ***)	0.47 *** (0.49 ***)	0.63 *** (0.60 ***)	1	0.39 *** (0.43 ***)	0.65 *** (0.60 ***)	0.45 *** (0.40 ***)	0.60 *** (0.59 ***)
6. Hostility	0.35 *** (0.44 ***)	0.39 *** (0.39 ***)	0.36 *** (0.42 ***)	0.39 *** (0.43 ***)	0.39 *** (0.43 ***)	1	0.26 *** (0.20 ***)	0.46 *** (0.52 ***)	0.41 *** (0.49 ***)
7. Phobic anxiety	0.51 *** (0.40 ***)	0.45 *** (0.48 ***)	0.54 *** (0.35 ***)	0.48 *** (0.40 ***)	0.65 *** (0.60 ***)	0.26 *** (0.20 **)	1	0.50 *** (0.29 ***)	0.52 *** (0.44 ***)
8. Paranoid ideation	0.41 *** (0.27 ***)	0.53 *** (0.45 ***)	0.70 *** (0.66 ***)	0.55 *** (0.45 ***)	0.45 *** (0.40 ***)	0.46 *** (0.52 ***)	0.50 *** (0.29 ***)	1	0.65 *** (0.58 ***)
9. Psychoticism	0.49 *** (0.47 ***)	0.58 *** (0.63 ***)	0.67 *** (0.67 ***)	0.65 *** (0.63 ***)	0.60 *** (0.59 ***)	0.41 *** (0.49 ***)	0.52 *** (0.44 ***)	0.65 *** (0.58 ***)	1

Note: ** *p* < 0.05 and *** *p* < 0.001.

**Table A2:** Centrality measures for each node across the three networks (global, individuals with suicidal ideation, and individuals without suicidal ideation).

Symptom Dimension	Group	Strength	Closeness	Betweenness	Expected Influence
Somatization	Nonsuicidal	0.85	0.02	1.79	0.74
	Suicidal	0.85	0.02	5.37	0.72
	Global	0.76	0.01	12.00	0.75
Obsessive-Compulsive	Nonsuicidal	0.83	0.02	0.90	0.81
	Suicidal	1.00	0.02	6.28	0.99
	Global	0.90	0.01	0.00	0.91
Interpersonal Sensitivity	Nonsuicidal	1.32	0.02	11.75	1.06
	Suicidal	1.06	0.02	9.14	0.99
	Global	1.05	0.01	8.00	1.06
Depression	Nonsuicidal	1.07	0.02	5.90	1.02
	Suicidal	0.92	0.02	3.86	0.9
	Global	0.96	0.01	14.00	0.97
Anxiety	Nonsuicidal	1.41	0.02	12.96	1.23
	Suicidal	1.17	0.02	12.80	1.17
	Global	1.23	0.01	22.00	1.24
Hostility	Nonsuicidal	0.60	0.01	0.11	0.45
	Suicidal	0.70	0.01	3.71	0.62
	Global	0.63	0.01	0.00	0.63
Phobic Anxiety	Nonsuicidal	0.83	0.02	3.71	0.65
	Suicidal	0.60	0.01	0.50	0.51
	Global	0.60	0.01	0.00	0.57
Paranoid Ideation	Nonsuicidal	1.03	0.02	7.92	0.97
	Suicidal	0.84	0.02	5.16	0.78
	Global	0.87	0.01	4.00	0.87
Psychoticism	Nonsuicidal	0.97	0.02	2.75	0.95
	Suicidal	1.02	0.02	4.52	1.02
	Global	1.11	0.01	10.00	1.12
Suicide	Global	0.46	0.01	2.00	0.45

**Table A3:** Bootstrap edge weights (global).

Node 1	Node 2	Edge Weight (Mean)	SE	*p*
Phobic Anxiety	Paranoid Ideation	−0.009	0.027	0.741
Phobic Anxiety	Psychoticism	−0.031	0.033	0.339
Anxiety	Phobic Anxiety	0.232	0.048	<0.001
Anxiety	Hostility	−0.025	0.045	0.573
Anxiety	Paranoid Ideation	−0.128	0.037	<0.001
Anxiety	Psychoticism	−0.003	0.023	0.897
Depression	Phobic Anxiety	−0.170	0.045	<0.001
Depression	Anxiety	−0.043	0.050	0.39
Depression	Hostility	−0.066	0.056	0.239
Depression	Paranoid Ideation	−0.136	0.041	<0.001
Depression	Psychoticism	−0.017	0.031	0.579
Hostility	Phobic Anxiety	−0.156	0.044	<0.001
Hostility	Paranoid Ideation	0.139	0.041	<0.001
Hostility	Psychoticism	0.007	0.034	0.842
Paranoid Ideation	Psychoticism	0.070	0.039	0.069
Obsessive-Compulsive	Phobic Anxiety	−0.058	0.034	0.084
Obsessive-Compulsive	Anxiety	−0.057	0.043	0.186
Obsessive-Compulsive	Depression	0.025	0.037	0.5
Obsessive-Compulsive	Hostility	−0.076	0.043	0.077
Obsessive-Compulsive	Paranoid Ideation	−0.095	0.037	0.011
Obsessive-Compulsive	Psychoticism	−0.041	0.033	0.213
Somatization	Phobic Anxiety	−0.069	0.042	0.106
Somatization	Anxiety	0.147	0.059	0.013
Somatization	Depression	−0.141	0.058	0.015
Somatization	Hostility	−0.046	0.046	0.314
Somatization	Paranoid Ideation	−0.121	0.038	0.001
Somatization	Obsessive-Compulsive	−0.138	0.045	0.002
Somatization	Psychoticism	−0.143	0.043	<0.001
Somatization	Interpersonal Sensitivity	−0.272	0.041	<0.001
Interpersonal Sensitivity	Phobic Anxiety	0.019	0.032	0.546
Interpersonal Sensitivity	Anxiety	−0.151	0.045	<0.001
Interpersonal Sensitivity	Depression	0.035	0.042	0.403
Interpersonal Sensitivity	Hostility	−0.099	0.043	0.02
Interpersonal Sensitivity	Paranoid Ideation	0.243	0.038	<0.001
Interpersonal Sensitivity	Psychoticism	0.057	0.040	0.152

Note: Edge weights represent the estimated partial correlations between nodes. Standard errors and *p*-values were derived from nonparametric bootstrap resampling (1000 samples); SE = standard error, *p* = significance.

**Table A4:** Bootstrap edge weights (nonsuicidal network, n = 394).

Node 1	Node 2	Edge Weight (Mean)	SE	*p*
Phobic Anxiety	Paranoid Ideation	0.103	0.058	0.075
Phobic Anxiety	Psychoticism	0.040	0.039	0.313
Anxiety	Phobic Anxiety	0.355	0.049	<0.001
Anxiety	Hostility	0.078	0.048	0.101
Anxiety	Paranoid Ideation	−0.035	0.045	0.434
Anxiety	Psychoticism	0.169	0.044	<0.001
Depression	Phobic Anxiety	−0.022	0.037	0.56
Depression	Anxiety	0.159	0.047	<0.001
Depression	Hostility	0.035	0.039	0.377
Depression	Paranoid Ideation	0.014	0.030	0.651
Depression	Psychoticism	0.158	0.052	0.002
Hostility	Phobic Anxiety	−0.059	0.050	0.239
Hostility	Paranoid Ideation	0.215	0.045	<0.001
Hostility	Psychoticism	0.050	0.048	0.3
Paranoid Ideation	Psychoticism	0.244	0.049	<0.001
Obsessive-Compulsive	Phobic Anxiety	0.008	0.024	0.74
Obsessive-Compulsive	Anxiety	0.084	0.047	0.075
Obsessive-Compulsive	Depression	0.300	0.045	<0.001
Obsessive-Compulsive	Hostility	0.087	0.049	0.077
Obsessive-Compulsive	Paranoid Ideation	0.033	0.037	0.374
Obsessive-Compulsive	Psychoticism	0.071	0.048	0.144
Obsessive-Compulsive	Interpersonal Sensitivity	0.212	0.047	<0.001
Somatization	Phobic Anxiety	0.053	0.044	0.234
Somatization	Anxiety	0.455	0.043	<0.001
Somatization	Depression	0.148	0.053	0.005
Somatization	Hostility	0.059	0.049	0.23
Somatization	Paranoid Ideation	0.026	0.034	0.449
Somatization	Obsessive-Compulsive	0.030	0.039	0.446
Somatization	Psychoticism	0.019	0.035	0.585
Somatization	Interpersonal Sensitivity	−0.050	0.046	0.283
Interpersonal Sensitivity	Phobic Anxiety	0.182	0.055	0.001
Interpersonal Sensitivity	Anxiety	−0.071	0.049	0.145
Interpersonal Sensitivity	Depression	0.228	0.052	<0.001
Interpersonal Sensitivity	Hostility	−0.011	0.031	0.72
Interpersonal Sensitivity	Paranoid Ideation	0.352	0.043	<0.001
Interpersonal Sensitivity	Psychoticism	0.209	0.052	<0.001

Note: Edge weights represent the estimated partial correlations between nodes. Standard errors and p-values were derived from nonparametric bootstrap resampling (1000 samples); SE = standard error, *p* = significance.

**Table A5:** Bootstrap edge weights (suicidal network, n = 203).

Node 1	Node 2	Edge Weight (Mean)	SE	*p*
Phobic Anxiety	Paranoid Ideation	0.017	0.037	0.648
Phobic Anxiety	Psychoticism	0.072	0.059	0.22
Anxiety	Phobic Anxiety	0.325	0.066	<0.001
Anxiety	Hostility	0.062	0.057	0.273
Anxiety	Paranoid Ideation	0.008	0.024	0.73
Anxiety	Psychoticism	0.116	0.056	0.038
Depression	Phobic Anxiety	0.011	0.031	0.733
Depression	Anxiety	0.107	0.058	0.066
Depression	Hostility	0.043	0.044	0.329
Depression	Paranoid Ideation	0.002	0.024	0.929
Depression	Psychoticism	0.155	0.060	0.009
Hostility	Phobic Anxiety	−0.042	0.061	0.49
Hostility	Paranoid Ideation	0.251	0.070	<0.001
Hostility	Psychoticism	0.122	0.064	0.058
Paranoid Ideation	Psychoticism	0.160	0.061	0.008
Obsessive-Compulsive	Phobic Anxiety	0.097	0.058	0.091
Obsessive-Compulsive	Anxiety	0.174	0.065	0.007
Obsessive-Compulsive	Depression	0.279	0.056	<0.001
Obsessive-Compulsive	Hostility	0.007	0.025	0.796
Obsessive-Compulsive	Paranoid Ideation	0.015	0.037	0.683
Obsessive-Compulsive	Psychoticism	0.128	0.052	0.014
Obsessive-Compulsive	Interpersonal Sensitivity	0.168	0.053	0.002
Somatization	Phobic Anxiety	0.019	0.035	0.592
Somatization	Anxiety	0.359	0.049	<0.001
Somatization	Depression	0.098	0.063	0.122
Somatization	Hostility	0.155	0.067	0.02
Somatization	Paranoid Ideation	−0.028	0.048	0.563
Somatization	Obsessive-Compulsive	0.126	0.069	0.069
Somatization	Psychoticism	0.032	0.043	0.459
Somatization	Interpersonal Sensitivity	−0.039	0.058	0.503
Interpersonal Sensitivity	Phobic Anxiety	0.010	0.029	0.721
Interpersonal Sensitivity	Anxiety	0.014	0.029	0.628
Interpersonal Sensitivity	Depression	0.213	0.062	<0.001
Interpersonal Sensitivity	Hostility	0.012	0.031	0.692
Interpersonal Sensitivity	Paranoid Ideation	0.358	0.056	<0.001
Interpersonal Sensitivity	Psychoticism	0.242	0.048	<0.001

Note: Edge weights represent the estimated partial correlations between nodes. Standard errors and *p*-values were derived from nonparametric bootstrap resampling (1000 samples); SE = standard error, *p* = significance.

**Table A6:** Summary of multicollinearity analysis (n = 621).

N = 621	Standardized Coefficients β	t	Sig.	Collinearity Statistics	
				Tolerance	VIF
Sex	−0.036	−0.950	0.342	0.922	1.085
Age	0.002	1.597	0.111	0.916	1.091
Somatization	−0.071	−2.192	0.029	0.421	2.375
Obsessive-Compulsive	0.002	0.055	0.956	0.407	2.456
Interpersonal Susceptibility	0.036	1.124	0.262	0.338	2.962
Depression	0.019	7.549	9.573	0.000	0.517
Anxiety	0.028	−0.256	0.760	0.448	0.313
Hostility	0.127	3.052	5.188	0.000	0.630
Phobic Anxiety	0.001	−0.680	0.033	0.974	0.527
Paranoid Ideation	−0.050	−1.179	−1.578	0.115	0.422
Psychoticism	0.028	1.695	0.091	0.318	3.141

Note. N = sample size; β = standardized coefficient; t = t-value; Sig. = significance level (*p*-value); VIF = variance inflation factor; *p* values indicate significance levels.
